# Crebl2 regulates cell metabolism in muscle and liver cells

**DOI:** 10.1038/s41598-019-56407-w

**Published:** 2019-12-27

**Authors:** Marcel Tiebe, Marilena Lutz, Deniz Senyilmaz Tiebe, Aurelio A. Teleman

**Affiliations:** 1 German Cancer Research Center (DKFZ), 69120 Heidelberg, Germany; 2 Heidelberg University, 69120 Heidelberg, Germany; 3 CellNetworks - Cluster of Excellence, Heidelberg University, Heidelberg, Germany

**Keywords:** Transcription, Metabolism

## Abstract

We previously identified Drosophila REPTOR and REPTOR-BP as transcription factors downstream of mTORC1 that play an important role in regulating organismal metabolism. We study here the mammalian ortholog of REPTOR-BP, Crebl2. We find that Crebl2 mediates part of the transcriptional induction caused by mTORC1 inhibition. In C2C12 myoblasts, Crebl2 knockdown leads to elevated glucose uptake, elevated glycolysis as observed by lactate secretion, and elevated triglyceride biosynthesis. In Hepa1-6 hepatoma cells, Crebl2 knockdown also leads to elevated triglyceride levels. In sum, this works identifies Crebl2 as a regulator of cellular metabolism that can link nutrient sensing via mTORC1 to the metabolic response of cells.

## Introduction

Metabolic pathways need to be tightly regulated to match a cell’s metabolic demands to the nutrients and energy supply provided by the environment. Since metabolic pathways are highly complex networks of enzymes with many components, their regulation is often controlled by transcription factors that have the potential to activate or repress multiple genes at the same time. One of those transcription factors is cAMP response element binding protein (CREB)^1^. CREB, among other functions, adapts metabolic demand to energy supply during starvation^1–4^ and has been implicated in diabetes^5,6^. For instance, CREB is activated upon starvation to promote gluconeogenesis and lipid oxidation in the liver^2,3^. One distinguishing characteristic of CREB is its basic leucine zipper (bZIP) domain that allows CREB to bind to CREs (cAMP responsive elements)^7–11^. Based on this domain, several CREB-like transcription factors have been identified that are involved in a variety of processes^6,7,12,13^. Previously, we identified two such CREB-like factors REPTOR (CG13624) and REPTOR-BP (CG18619) as transcription factors downstream of mTORC1 that play an important role in regulating metabolism in *Drosophila*^14^. We reported that when mTORC1 is active, it phosphorylates REPTOR, leading to its cytoplasmic retention. When mTORC1 activity drops, REPTOR enters the nucleus where it binds REPTOR-BP to induce transcription of a battery of genes that regulate fat metabolism, glycogen metabolism, and the response of adult flies to starvation. In the absence of REPTOR or REPTOR-BP, Drosophila are very lean and extremely sensitive to starvation^14^.

The mammalian orthologs of REPTOR and REPTOR-BP are Crebrf and Crebl2, respectively. In mice, Crebrf was first described as LRF (Luman recruiting factor) based on the observation that LRF can bind Luman and suppress its function in the unfolded protein response (UPR)^15^. Crebrf KO female mice show a lack of instinct to tend their pups and reduced prolactin levels^16^. In this context, Crebrf was found to bind to and repress the glucocorticoid receptor, thereby regulating lactating behavior^16^. Recently, an allele of Crebrf (p.Arg457Gln) was linked to increased BMI and protection from diabetes in Pacific populations of Polynesia^17–20^. Indeed, this SNP in Crebrf has an effect size that is much larger than that of any other known common BMI risk variant^17,21–23^. Hence, like its fly homolog, Crebrf also regulates metabolic traits in humans.

In contrast to Crebrf, the role of Crebl2 in regulating cellular or organismal metabolism has been less studied. Crebl2 knockout mice have not been reported thus far, however 3T3-L1 cells lacking Crebl2 fail to differentiate into adipocytes^24^. Conversely, overexpression of Crebl2 was sufficient to drive adipogenesis in these cells, and Crebl2 was also shown to bind CREB in this context^24^. Similar to CREB, Crebl2 can also be phosphorylated by AMPK^25^. Hence Crebl2 plays a role in adipocyte differentiation.

We study here the role of Crebl2 in regulating cellular metabolism. Like its Drosophila ortholog, we find that Crebl2 binds Crebrf. We find that loss of Crebl2 has strong consequences on cellular metabolism in both myocytes and hepatocytes, where it regulates glucose uptake and lipid accumulation. In sum, this work identifies Crebl2 as a regulator of cellular metabolism and suggests that Crebl2 knockout mice would be worth studying in the future.

## Results

### Crebl2 is required for part of the transcriptional response caused by mTORC1 inhibition in MEFs

**Figure 1:**
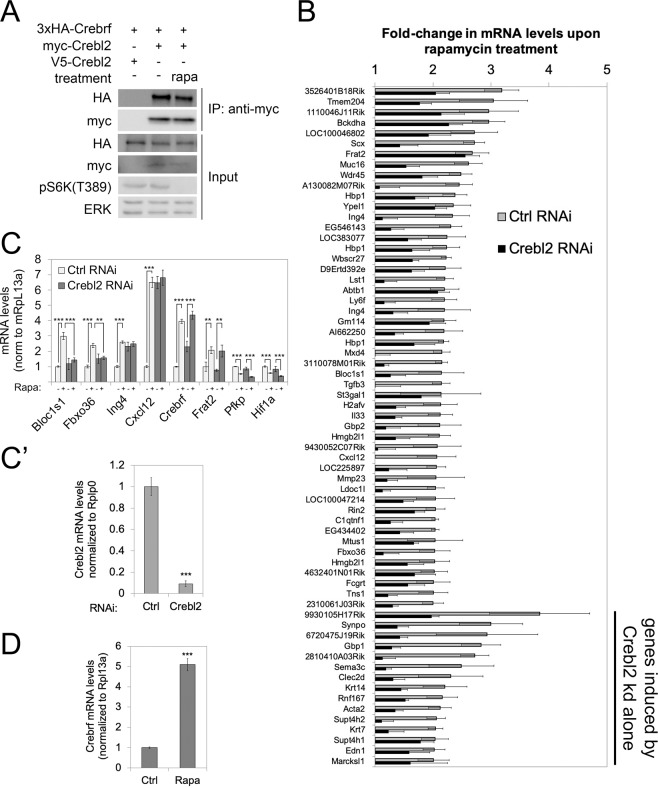
Rapamycin induced transcription is partially dependent on Crebl2 in MEFs. (**A**) Crebl2 and Crebrf bind each other. Co-IP of 3xHA-CREBRF with myc-CREBL2 in HEK293T cells. V5-Crebl2 is used as a negative control for the myc-IP. (**B**) Crebl2 is required for induction of gene expression in response to rapamycin (20 nM, 12 h). Fold change after rapamycin treatment from Illumina BeadChip analysis for the top inducing genes in control (grey) and Crebl2 knockdown (black) TSC2^−/−^ MEFs. Error bars represent biological triplicates except Luciferase control knockdown samples which were duplicates. (**C**,C’) Knockdown of Crebl2 prevents rapamycin (20 nM, 12 h) induction of target genes in TSC2^−/−^ MEFs. mRNA levels by qPCR, normalized to Rpl13a. Frat2 is shown as unaffected control and Pfkp, Hif1a are shown as rapamycin-repressed controls. Knockdown efficiency of Crebl2 shown in (C’). Error bars represent technical triplicates. ***p < 0.001, **p < 0.01 by two-way ANOVA. (**D**) Crebrf is transcriptionally induced by rapamycin treatment (20 nM, 12 h) in *TSC2*^−/−^ Mefs. mRNA levels by qRT-PCR, normalized to Rpl13a. Error bars represent technical triplicates. ***p < 0.001 by t-test. All panels: error bars = SD.

Since *Drosophila* REPTOR and REPTOR-BP form a transcriptional complex, we first tested whether the human orthologs Crebrf and Crebl2 can also bind each other. Indeed, myc-tagged Crebl2 was able to co-immunoprecipitate (coIP) HA-tagged Crebrf (Fig. 1A). Since the Drosophila orthologs are regulated by mTORC1 and control transcription downstream of mTORC1, we next tested whether Crebl2 also mediates part of the transcriptional response caused by mTORC1 inhibition. To this end, we measured genome-wide expression levels of mRNAs in TSC2(−/−) mouse embryonic fibroblasts (MEFs) after 12 hours of rapamycin or vehicle treatment in the presence or absence of a Crebl2 knockdown. These cells have been used previously to study the transcriptional response to rapamycin because they provide a good dynamic range for the response^26–29^. We thereby identified 61 genes with a significant increase in expression of at least 2-fold upon rapamycin treatment in control cells (grey bars, Fig. 1B, and Suppl. Tables 1 and 2). Knockdown of Crebl2 blunted the induction of the majority of these genes (black bars, Fig. 1B), indicating that Crebl2 is required for the appropriate activation of gene expression caused by mTORC1 inhibition. We confirmed these results by quantitative RT-PCR on additional biological replicates (Fig. 1C,C’). Some target genes such as Bloc1s1 or Fbxo36 were not affected in their baseline levels by Crebl2 knockdown but lost their induction in response to rapamycin treatment (Fig. 1C). Other targets such as Cxcl12 or Ing4, which require Crebl2 for their induction in response to rapamycin treatment, already start with elevated expression in the control condition (Fig. 1C). We observed this for 15 of the 61 induced genes (Fig. 1B), suggesting these genes may be part of more complex transcriptional networks that adapt in response to Crebl2 loss. In contrast, genes such as Pfkp or Hif1a, which are repressed by rapamycin treatment^29^, were unaffected by Crebl2 knockdown (Fig. 1C). Thus, similar to its *Drosophila* ortholog REPTOR-BP, Crebl2 is required in mouse embryonic fibroblasts for part of the transcriptional induction caused by mTORC1 inhibition.

### Crebrf is transcriptionally induced upon mTORC1 inhibition

The data presented above suggest that Crebl2 may function downstream of mTORC1. Alternatively, Crebl2 could function in parallel and independently of mTORC1 but be required for mTORC1 to regulate gene expression. To test the first option, we asked whether the interaction between Crebl2 and Crebrf, or their localization, is regulated by mTORC1, as is the case for the Drosophila orthologs^14^. Rapamycin treatment, however, did not affect the amount of Crebrf co-immunoprecipitating with Crebl2 (Fig. 1A). To analyze the subcellular localization of Crebrf and Crebl2, we tested multiple commercially available antibodies for each protein, but could not find any that gave a specific signal in MEFs, Hepa1-6 cells or C2C12 cells. Despite experience in generating antibodies ourselves, we also failed to generate antibodies against either protein. Hence, instead, we analyzed the subcellular localization of epitope tagged Crebrf or Crebl2. Previous reports pointed to a strictly nuclear speckled localization of overexpressed, epitope-tagged human Crebrf^15,16^. We found that N-terminally HA-tagged Crebrf was either nuclear or cytoplasmic or both in TSC2−/− MEFs, and this varied from cell to cell (Suppl. Fig. 1A). In HEK293T cells HA-Crebrf was mostly cytoplasmic (Suppl. Fig. 1B) whereas GFP-tagged Crebrf was mostly nuclear (Suppl. Fig. 1C). Since the localization of Crebrf differed between cells within one population and was dependent on the identity of the tag, we could not confidently assess Crebrf subcellular localization. This also suggests that previous data regarding Crebrf subcellular localization based on overexpressed tagged protein may need to be interpreted with caution. Likewise, to assess the subcellular localization of Crebl2 we expressed HA, V5 or myc-tagged Crebl2 in TSC2−/− MEFs. Whereas V5-Crebl2 was exclusively nuclear (Suppl. Fig. 1D), HA-Crebl2 was cytoplasmic in some cells and nuclear in other cells (Suppl. Fig. 1E) and myc-Crebl2 formed speckles in the nucleus (Suppl. Fig. 1F). In sum, we could not conclude anything regarding the subcellular localization of Crebrf or Crebl2.

In line with previous data^17,29^, however, we observed that Crebrf is transcriptionally induced upon rapamycin treatment (Fig. 1D), also if Crebl2 is knocked-down (Fig. 1C). Interestingly, the Crebrf ortholog REPTOR is also activated upon rapamycin treatment in *Drosophila*, albeit by a change in cellular localization^14^, pointing to a conserved activation of REPTOR and Crebrf upon mTORC1 inhibition. In sum, the Crebrf/Crebl2 complex in Drosophila and mammalian cells mediates part of the transcriptional response to mTORC1 inhibition, and is transcriptionally induced upon mTORC1 inhibition, suggesting it is likely functioning downstream of mTORC1.

### Crebl2 regulates lipid metabolism in myoblasts and hepatocytes

**Figure 2:**
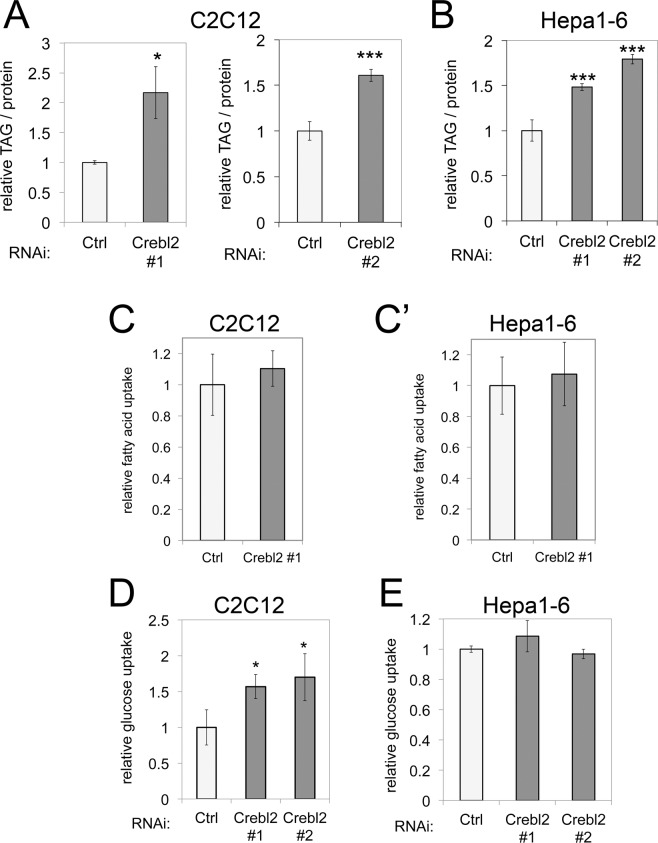
Crebl2 knockdown increases fat content in mouse myoblasts and hepatoma cells. (**A**) Knockdown of Crebl2 increases triglyceride (TAG) content of C2C12 myoblasts. C2C12 were treated with control or two independent siRNAs against Crebl2 for 3 days before TAG measurement, normalized to total cell protein. (n = 3). (**B**) Knockdown of Crebl2 increases triglyceride content of Hepa1-6 hepatoma cells. Hepa1-6 cells were treated with control or two independent siRNAs against Crebl2 for 3 days before fat measurement, normalize to total cell protein. (n = 3). (**C**,**C’**) Knockdown of Crebl2 does not affect lipid uptake of C2C12 or Hepa1-6 hepatoma cells. Hepa1-6 cells were treated with control or siRNAs against Crebl2 for 3 days before lipid uptake assay. (n = 3). (**D**) Knockdown of Crebl2 increases glucose uptake in C2C12 myoblasts. C2C12 myoblasts were treated with control or two independent siRNAs against Crebl2 for 3 days before glucose uptake assay, normalized to total cell protein. (n = 3). (**E**) Knockdown of Crebl2 does not affect glucose uptake of Hepa1-6 cells. Hepa1-6 cells were treated with control or two independent siRNAs against Crebl2 for 3 days before glucose uptake assay, normalized to total cell protein. (n = 3). Error bars: SD ***t-test < 0.001, **t-test < 0.01, *t-test < 0.05.

Since both the Crebl2 interaction with Crebrf and its transcriptional function downstream of mTORC1 appear to be conserved in Drosophila and mammalian cells, we next tested whether Crebl2, similar to REPTOR-BP, also affects cellular metabolism. For that purpose, we used two cell lines, C2C12 myoblasts and Hepa1-6 hepatoma cells, representing two of the main metabolic tissues in mammals. Importantly, in both cell lines Crebrf was also induced upon rapamycin treatment similar to TSC2−/− Mefs (Supp. Fig. 2A). In fact, Crebrf was one of only 5 genes (Lst1, Krt14, Ypel1, Frat2, Edn1) that were induced by rapamycin in both C2C12 myoblasts and TSC2−/− MEFs, suggesting a general role of Crebrf/Crebl2 upon mTORC1 inhibition. We also measured changes in gene expression after rapamycin treatment in C2C12 cells with or without Crebl2 knockdown via RNA-Seq. While in control cells 276 genes are transcriptionally activated in C2C12 cells, only 107 of those fulfill the induction criteria (2-fold induction after rapamycin treatment, p < 0.05) in cells lacking Crebl2 (Suppl. Table 3), suggesting that Crebl2 is also required for the proper transcriptional response to mTORC1 in C2C12 myoblasts. We therefore knocked down Crebl2 in C2C12 and Hepa1-6 cells (Suppl. Fig. 2B,C) and tested for metabolic phenotypes similar to the ones we found in flies. When knocking down Crebl2 using two independent siRNAs in mouse C2C12 myoblasts we observed a strong increase in triglyceride (TAG) content of these cells (Fig. 2A). Likewise, Crebl2 knockdown led to a significant increase in TAG content in Hepa1-6 cells (Fig. 2B). Since the BSA in serum contains fatty acids, one possible explanation for this phenotype is that Crebl2 knockdown causes cells to uptake more lipids from the medium. This was not the case, however, as observed by providing cells with fluorescent palmitic acid and assaying uptake by flow cytometry (Fig. 2C,C’). Alternatively, elevated TAG levels can result from elevated fatty acid biosynthesis using glucose as a carbon source, or from reduced beta-oxidation. We observed a 50% increase in glucose uptake upon Crebl2 knockdown in C2C12 cells (Fig. 2D) but not in Hepa1–6 cells (Fig. 2E). Hence, although Crebl2 affects lipid metabolism in both cell types, it appears to do so differently in muscle versus liver cells. Thus we focused our attention on C2C12 cells for follow-up experiments.

### Loss of Crebl2 hyperactivates energy production and storage pathways in C2C12 cells

**Figure 3:**
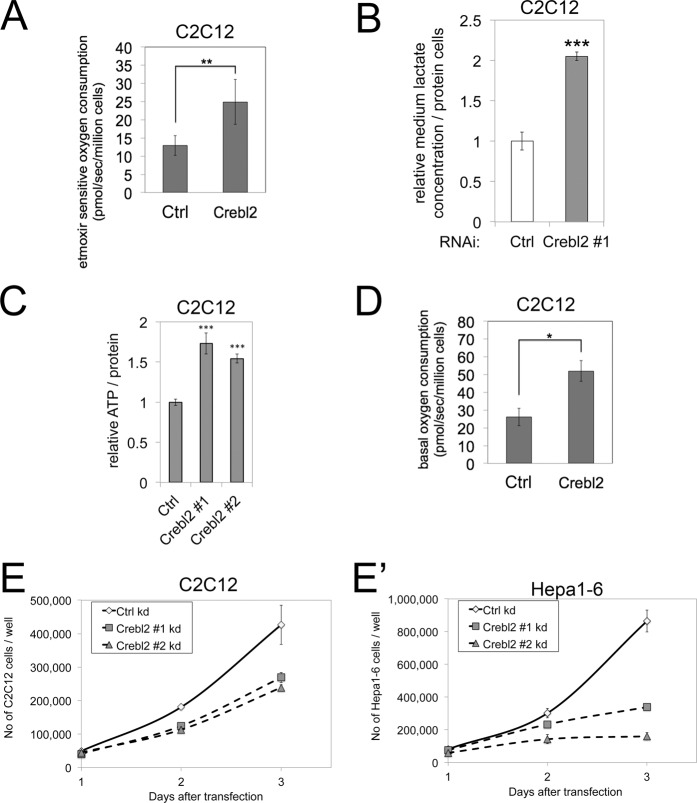
Crebl2 knockdown disrupts catabolic metabolism in C2C12 myoblasts. (**A**) Knockdown of Crebl2 increases beta-oxidation in C2C12 cells. After 2.5 days of siRNA transfection, beta-oxidation was measured as the etomoxir-dependent oxygen consumption using an Oroboros Oxygraph. (n = 4). (**B**) Knockdown of Crebl2 increases lactate secretion of C2C12 cells. Cells were transfected with siRNA for 2.5 days, medium was removed and replaced with fresh medium and lactate concentration in the medium was measured after 2 hours of conditioning, normalized to cellular protein content. (n = 2-3). (**C**) ATP levels are increased upon 3 days of Crebl2 knockdown in C2C12 myoblasts. (n = 3). (**D**) Knockdown of Crebl2 increases basal oxygen-consumption in C2C12 cells. After 2.5 days of siRNA transfection, basal oxygen consumption was measured in untreated, unpermeabilized cells using an Oroboros Oxygraph. (n = 4). (**E**,E’) Knockdown of Crebl2 decreases proliferation rate of C2C12 and Hepa1–6 cells. 1 day after siRNA transfection either C2C12 (**E**) or Hepa1–6 (E’) cells were counted every day for three days. (n = 3). Error bars: SD ***t-test < 0.001, **t-test < 0.01, *t-test < 0.05.

Since fatty acid uptake is not elevated in Crebl2 knockdown cells, the increased TAG levels can result either from increased FA biosynthesis or reduced FA beta-oxidation. We therefore measured beta-oxidation levels in C2C12 cells by measuring cellular oxygen consumption in the presence or absence of etomoxir, which blocks CPT1-dependent fatty acid uptake into mitochondria. Surprisingly, Crebl2 knockdown cells have elevated beta-oxidation rates compared to control cells (Fig. 3A), indicating that Crebl2 knockdown cells must have very high FA biosynthesis rates leading to elevated TAG levels despite the elevated beta-oxidation. The carbon atoms entering a cell as glucose can partly be used for fatty acid biosynthesis, partly be secreted as lactate at the end of glycolysis, and partly be oxidized to CO_2_ by the TCA cycle for mitochondrial respiration. Hence there is often a balance between these carbon fluxes. We therefore measured lactate secretion and unexpectedly found that Crebl2 knockdown cells have elevated lactate secretion (Fig. 3B). We also measured cellular ATP levels and found those are also elevated in Crebl2 knockdown cells (Fig. 3C), as is basal oxygen consumption (Fig. 3D). Altogether, this implies that the increased glucose uptake caused by Crebl2 knockdown in C2C12 cells is sufficient to simultaneously fuel elevated lactate secretion, elevated fatty acid biosynthesis, and elevated respiration.

### Crebl2 knockdown cells have reduced proliferation rates

The metabolic phenotype described above is peculiar because it is a combination of increased energy storage (TAGs) and increased energy release (beta oxidation, glycolysis) whereas cells usually antagonistically regulate anabolic and catabolic pathways. We hypothesized this may happen at the expense of biomass accumulation, resulting in impaired proliferation rates. Indeed both C2C12 and Hepa1–6 cells showed a strong reduction in proliferation when Crebl2 was knocked down (Fig. 3E,E’).

### Transcriptional consequences of Crebl2 knockdown in C2C12 and Hepa1–6 cells

**Figure 4:**
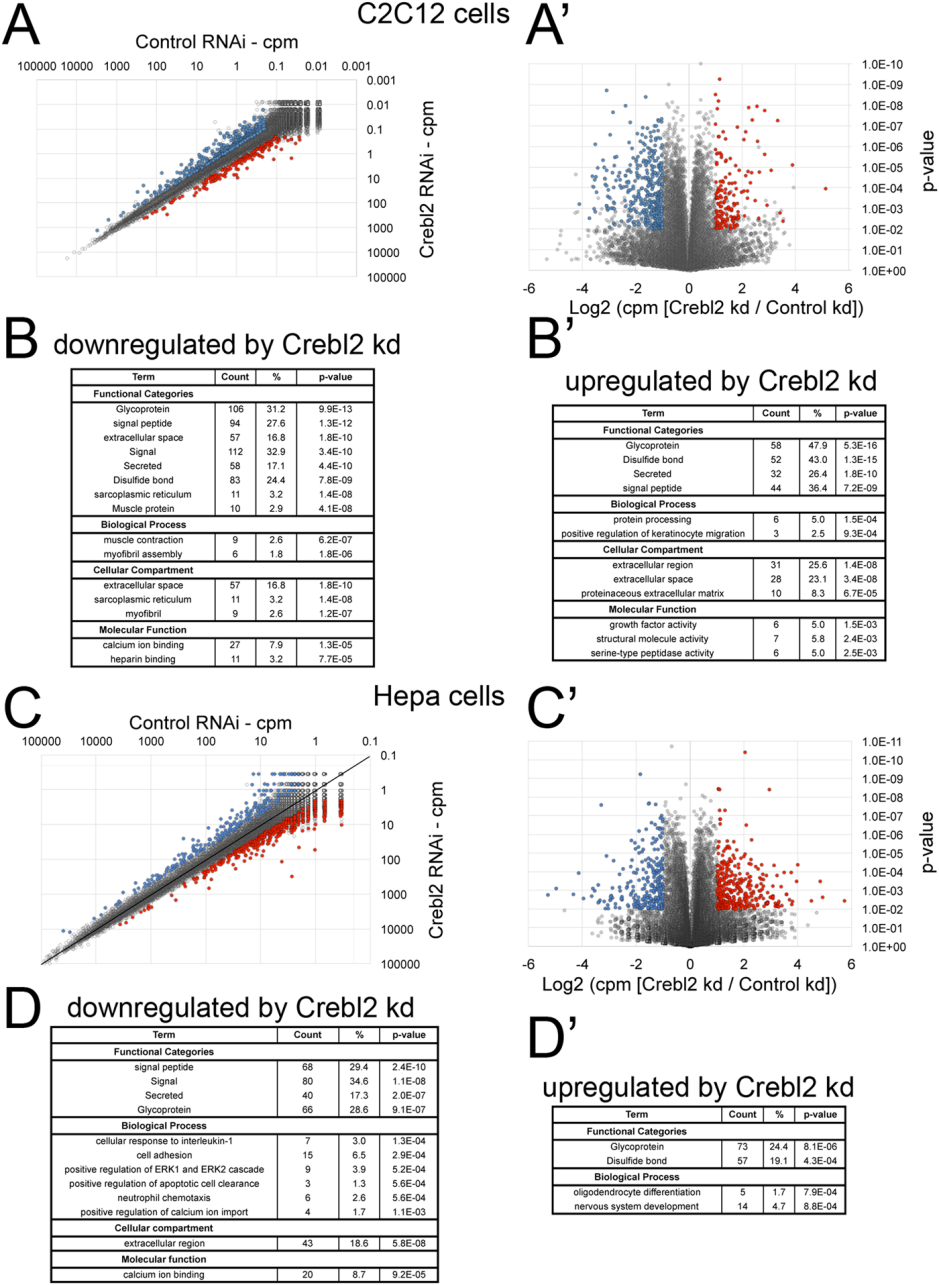
Gene expression profiles upon Crebl2 knockdown in C2C12 and Hepa1–6 cells. (**A,B**) Expression profile of C2C12 myoblasts lacking Crebl2. C2C12 cells were treated with control siRNA or siRNA against Crebl2 for 3 days before RNA extraction and RNA-Seq analysis. Expression of each gene is given as counts per million reads (cpm) and compared between control and Crebl2 knockdown (**A**) and shown as a volcano blot (**A’**). Genes that are significantly down-regulated (**B**) or up-regulated (**B’**) upon Crebl2 knockdown are enriched in secreted proteins, glycoproteins and muscle proteins. (**C,D**) Expression profile of Hepa1–6 cells lacking Crebl2. Hepa1–6 cells were treated with control siRNA or siRNA against Crebl2 for 3 days before RNA extraction and RNA-Seq analysis. Expression of each gene is given as counts per million reads (cpm) and compared between control and Crebl2 knockdown (**C**) and shown as volcano plot (**C’**). Genes that are significantly down-regulated (**D**) or up-regulated (**D’**) upon Crebl2 knockdown are enriched in secreted protein and glycoproteins.

To find Crebl2 transcriptional targets that mediate the increased fat content observed in both C2C12 and Hepa1–6 cells, we knocked down Crebl2 in both cell lines and performed genome-wide expression analysis via RNA-Seq. In C2C12 cells we found 408 genes downregulated and 164 genes upregulated upon Crebl2 knockdown (Fig. 4A,A’ and Suppl. Tables 4 and 5). Both upregulated and downregulated genes were enriched in glycoproteins and proteins that have a signal peptide (Fig. 4B,B’). In Hepa1–6 cells we found 267 genes downregulated and 396 genes upregulated upon Crebl2 knockdown (Fig. 4C,C’ and Suppl. Tables 6 and 7). Also here, both groups were enriched for genes that are classified as glycoproteins and the downregulated genes were enriched for proteins that have a signal peptide (Fig. 4D,D’).

**Figure 5:**
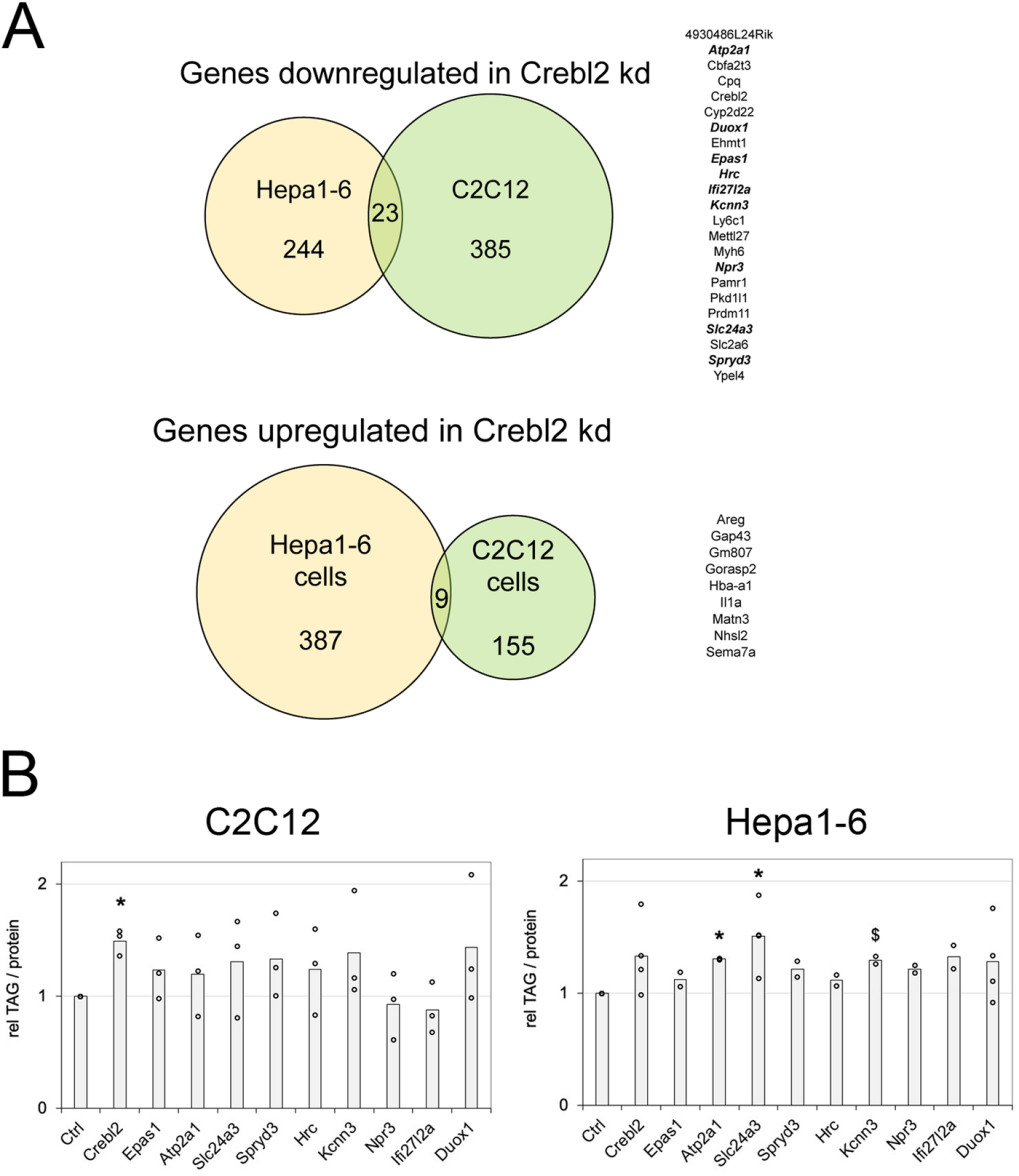
Common Crebl2 targets in myoblast and hepatoma cells. (**A**) Overlap of Crebl2 dependent genes in C2C12 myoblasts and Hepa1–6 cells. Genes in bold italic were tested for their effect on TAG levels in (**B**). (**B**) Summary of 7 different experiments (3 in C2C12, 4 in Hepa1–6 cells) comparing TAG levels of cells treated for 3 days with control and Crebl2 siRNA #1. Control cells were normalized to 1 for each experiment in order to combine and compare multiple biological replicates. *p < 0.05, ^$^p < 0.1 by one-sample two-tailed t-test with a hypothetical mean of 1.0.

Since both C2C12 and Hepa1–6 cells share a common phenotype of elevated TAG levels upon Crebl2 knockdown, we searched for genes that were misregulated in both cell lines. Surprisingly, we found only 23 genes (including Crebl2 itself) that are downregulated upon Crebl2 knockdown in both cell lines (Fig. 5A) and 9 genes that were up-regulated in both (Fig. 5A). This is likely because C2C12 and Hepa1–6 cells originate from different tissues and hence have different expression profiles. We screened the literature for any connection of these 32 genes to metabolism or metabolic regulation, and found 9, all amongst the down-regulated genes, which we chose for follow-up functional assays. We performed multiple rounds of testing in which we knocked down these 9 genes (or a subset thereof) in C2C12 cells or Hepa1–6 cells and quantified TAG levels in the cells (Fig. 5B). Interestingly, knockdown of 7 out of those 9 genes showed a tendency to increase cellular TAG levels (Fig. 5B). No single one, however, gave as strong a phenotype as the Crebl2 knockdown itself. Hence it is possible that Crebl2 knockdown causes increased TAG levels in C2C12 and Hepa1–6 cells via the combined reduction in expression of several genes.

## Discussion

This work identifies Crebl2 is a metabolic regulator. Knockdown of Crebl2 causes a strong lipid metabolism phenotype, leading to a doubling in cellular triglyceride (TAG) stores in C2C12 myoblasts and Hepa1–6 cells. Although Crebl2 regulates lipid metabolism in both C2C12 and Hepa1–6 cells, it does so somewhat differently in these two cell types. In C2C12 cells, Crebl2 knockdown leads to increased glucose uptake. This increased glucose uptake is sufficient to fuel more energy storage in the form of TAGs, as well as elevated glycolysis and lactate secretion. In Hepa1–6 cells, Crebl2 knockdown does not cause increased glucose uptake (Fig. 2E), nor does it lead to elevated lactate secretion (not shown). Hence it seems to only reroute glucose towards energy storage. One possible explanation for this difference could be that Crebl2 knockdown might enhance GLUT4 function leading to increased glucose uptake. Since GLUT4 is expressed in muscle cells but not in hepatocytes, which take up most glucose via GLUT2, this might explain why glucose uptake is unchanged in Hepa1–6 cells upon Crebl2 knockdown. Nonetheless, the increased TAG is a common phenotype in both cell types. Since Crebl2 knockout mice have not been reported, it will be interesting to study the physiological consequences of Crebl2 loss-of-function in an organismal setting. Indeed, a SNP near human Crebl2 was found to be significantly associated with the metabolic parameter Homeostasis Model Assessment of beta-cell function (HOMA-b)^30^.

Although Crebl2 regulates metabolism, it seems to do so in a tissue-specific manner. A previous report showed that in 3T3-L1 cells Crebl2 is required for adipogenesis. Knockdown of Crebl2 leads to a defect in adipocyte differentiation, and as a consequence impaired TAG accumulation^24^. Hence the final consequence of Crebl2 knockdown in adipocytes is the opposite to what we observe in muscle or liver cells, which is an increase in TAG levels. Furthermore, the genes we find dysregulated upon Crebl2 knockdown in C2C12 cells shows little overlap with the genes dysregulated in Hepa1–6 cells (Fig. 5A). These results highlight that metabolic regulation can be tissue specific, and in particular that Crebl2 likely has tissue specific effects that will add up in a complex manner at the organismal level.

We show here that Crebl2 and Crebrf can form a heteromeric complex, analogous to the one formed by their Drosophila orthologs^14^, suggesting they may be functioning together. Indeed, both Crebl2 and Crebrf promote adipogenesis^17,24^. Hence it is likely that Crebrf also plays a metabolic regulatory role in muscle and liver cells. Furthermore, Crebl2 also binds CREB^24^ hence Crebl2 may be acting as a cofactor for several metabolically relevant transcription factors of the CREB family.

In Drosophila we found that the orthologous proteins REPTOR and REPTOR-BP are regulated by mTORC1 activity. When mTORC1 activity is high, this leads to phosphorylation and cytosolic retention of REPTOR. When mTORC1 activity drops, REPTOR enters the nucleus where it binds REPTOR-BP and activates expression, thereby altering organismal metabolism^14^. There are both similarities and differences between the fly and mammalian proteins. Both Crebl2 and REPTOR-BP are required for the induction of most of the genes that are transcriptionally induced upon mTORC1 inhibition (Fig. 1B). Both the fly and human genes regulate metabolism. Both REPTOR and Crebrf are induced upon mTORC1 inhibition. However, in the case of REPTOR this happens via phosphorylation whereas for Crebrf this occurs at least in part via transcriptional induction. We cannot exclude that Crebrf might also be regulated by mTORC1 at the post-translational level. Unfortunately, we could not assess Crebrf subcellular localization because we were unsuccessful at generating Crebrf antibody despite several attempts, and the localization of epitope-tagged Crebrf depends on the tag that is used (Suppl. Fig. 1). Although we did not see relocalization of epitope-tagged Crebrf upon rapamycin treatment, this could be due to overexpression, or to interference by the epitope tags. Hence further work will be required to address this issue by detecting the endogenous protein.

We find circa 61 genes that are transcriptionally induced by rapamycin in MEFs, and the induction of many of these is blunted upon Crebl2 knockdown. These target genes can be grouped into two classes. Genes in the major class have low expression in the absence of Crebl2, and reduced induction in response to rapamycin, such as Bloc1s1. Genes in the second class have elevated basal expression upon Crebl2 knockdown and consequently blunted induction, such as Cxcl12 (Fig. 1C). One possible explanation for this second class of targets is that perhaps Crebl2 suppresses their expression, and this repression is alleviated by mTORC1 inhibition. However, in this case Crebl2 would need to be activated by mTORC1 (for the 2^nd^ class) and repressed by mTORC1 (for the 1^st^ class) which in our opinion makes this scenario unlikely. The second possible explanation is that Crebl2 always induces gene expression and is activated by rapamycin, but for some genes, basal expression levels increase upon loss of Crebl2 due to compensatory increases in other signaling pathways. Indeed, we have seen this in the past, for instance for FOXO where loss of FOXO leads to increased basal levels of the bona-fide target Lk6 rather than reduced levels of the induced state (Fig. 2D in^31^).

How does Crebl2 knockdown cause increased cellular TAG levels? We tested Crebl2 targets in both C2C12 and Hepa1–6 cells to see if any of them reproduces the high-TAG phenotype caused by Crebl2 knockdown (Fig. 5B). Although many showed a tendency towards increasing cellular TAG levels, none of them gave as strong and as consistent a phenotype as Crebl2 itself. Hence we believe most likely the phenotype caused by Crebl2 knockdown represents the combined sum of several different target genes. Surprisingly, the most enriched Gene Ontology category for both up-regulated and down-regulated transcripts upon Crebl2 knockdown in C2C12 cells are mRNAs having to do with protein secretion and glycosylation. Encoded by these mRNAs are both proteins involved in the secretory process, as well as proteins that are secreted themselves. The fact that some are up-regulated and some are down-regulated suggests that Crebl2 knockdown leads to a change in the cells’ secretome. The biological significance of this, and the relationship to metabolism, is unclear, and a topic for future study.

In sum, we show here that Crebl2 mediates part of the transcriptional response caused by mTORC1 inhibition and identify Crebl2 as a metabolic regulator.

## Materials and Methods

### Cell culture

TSC2^−/−^ p53^−/−^ MEFs, NIH3T3, C2C12 and Hepa1–6 cells were cultured in DMEM (4.5 g/L Glucose, L-Glutamine, #41965–039 GIBCO) with 10% FBS and Pen-Strep. TSC2−/−p53−/− MEFs were a kind.pngt by David Kwiatkowski and Michael Hall and were described previously^32^.

### Plasmids

All plasmids used are based on the pcDNA3.1 (+) vector, driving expression using the CMV promoter. pcDNA3.1(+) was modified by adding either N-terminal HA, 3xHA, GFP, V5 or myc tags between the BamHI and EcoRI site. Then human Crebl2 and Crebrf were cloned in-frame into these modified vectors using the EcoRI and NotI site. Crebl2 was cloned by using the oligos ggccgaattcATGGATGACAGTAAGGTGGTT (fw) and ggccgcggccgcTGGCTTCAATCACTGACTCA (rv). Crebrf was cloned by using ggccgaattcATGCCTCAGCCTAGTGTAAGC (fw) and ggccgcggccgcGGCTGATTACACCTTTGATGT (rv).

### Antibodies

Antibodies used were: mouse monoclonal anti-Myc tag (9b11) (Cell Signaling #2276) used at 1:1000 for immunoblot analysis, 1:400 for immunostainings and 1:500 for immunoprecipitation, rat monoclonal anti-HA (3F10) (Roche #11867423001) used at 1:1000 for immunoblot analysis and 1:400 for immunostainings, rabbit anti-pS6K (Thr389) (Cell Signaling #9205) used at 1:1000 for immunoblot analysis, rabbit monoclonal anti-ERK1/2 (Cell Signaling #4695) used at 1:2000 for immunoblot analysis.

### Immunoprecipitation and protein analysis

NIH3T3 cells were transfected with indicated plasmids using Lipofectamine 2000 (Thermo Fisher, #12566014) for 24 hours, lysed in IP lysis buffer (50 mM Tris pH7.5, 150 mM NaCl, 1% Triton-X with 2x protease inhibitor cocktail (Roche, 11836145001), 1x phosphatase inhibitor cocktail (Roche, 11836170001), Sodium vanadate (2 mM), Sodium fluoride (50 mM) and glycerol 2-phosphate (1 g/l)) for 30 minutes on ice. Cleared supernatant was incubated with antibody overnight, immunocomplexes were pulled down with ProteinA/G-beads and eluted with 2x Laemmli. Lysates were separated on an SDS-Polyacrylamide Gel and transferred on Nitrocellulose using a Wet Blot system.

### Immunostainings

Cells were fixed using 4% formaldehyde in 1xPBS for 20 min, blocked with 0.1% BSA and 0.2% TritonX-100 in 1xPBS, stained with indicated antibodies over night at 4 degree. After staining with secondary antibodies cells were counterstained with DAPI, Rhodamine-phalloidin or Cy5-phalloidin and then mounted in a glycerol-based mounting medium. Images were recorded using a Leica SP8 confocal system with a 63x or 40x objective with 1x or 2.5x digital magnification, depending on experiment. Scale bars are indicated in Figure legends.

### RNAi experiments

For RNAi experiments, the following Dharmacon siGENOME siRNAs were used: Crebl2-1 #D-053521-01 (Crebl2 RNAi #1, Target sequence GAGAGGAAC-UGGAAAUGUA), Crebl2-4 #D-053521-01 (Crebl2 RNAi #2, Target sequence CGGCAGAGUGCGAGAGAAU), Duox1 (pool) #D-047172-01/-02/-03/-04, Hrc (pool) #D-044598-01/-02/-03/-04, Kcnn3 (pool) #D-041893-01/-04/-17/-18, Ifi27l2a (pool) #D-063667-01/-02/-03/-04, Atp2a1 (pool) #D-064501-01/-02/-03/-04, Epas1 (pool) #D-040635-01/-02/-03/-04, Slc24a3 (pool) #D-040462-01/-02/-03/-04, Spryd3 (pool) #D-066303-13/14/15/16, Npr (pool) #D-047912-01/02/03/04. siRNA against Renilla Luciferase (Dharmacon, #P-002070-50, target sequence AAAAACATGCAGAAAAT-GCTG) was used as control. For transfection we used Lipofectamine RNAiMAX from Invitrogen (#13778). siRNA final concentration used for all cell lines is 20 nM.

### TAG assay

To measure cellular TAG levels, we lysed cells from one subconfluent 10 cm dish in 250 µl (Crebl2 kd samples) or 400 µl (Luciferase kd samples) lysis buffer (1xPBS with 0.05% Tween-20 (Applichem, A1389)) to achieve similar protein concentrations in both samples as Crebl2 grow significantly slower than control cells. Then, 3 µl of Lipase (10 mg/ml, Sigma, #437707), was added to 70 µl non-cleared lysate and incubated over night at 37 °C. Lysate was cleared by centrifugation and then 50 µl of lysate was incubated with 250 µl Free Glycerol reagent (Sigma, #F6428) for 5 min at 37 °C. Afterwards OD was measured at 540 nm. Values were normalized to protein content of lysates. Samples were measured in biological triplicates unless stated otherwise.

### Glucose uptake assay

After 3 days of knockdown, 100 µM 2-NBDG (Cayman, #11046) was added for 30 minutes directly to the growth medium. Cells were washed with fresh medium twice, then trypsinized and 2-NBDG uptake was quantified by flow cytometry. Samples were measured in biological triplicates unless stated otherwise.

### Lipid uptake assay

1:5000 dilution of BODIPY FL C_16_ solution (1 mg/ml in DMSO, #D3821 Thermo Fisher) was added for 5 minutes to C2C12 or Hepa1–6 cells and then washed away with PBS. Cells were trypsinized for 7 min and resuspended with 1% Fatty-Acid-Free BSA (SERVA, #11945), mixed, and then analyzed via flow cytometry. Samples were measured in biological triplicates unless stated otherwise.

### Lactate assay

To measure secreted lactate, we removed medium after 3 days of knockdown and added fresh medium. After 2 hours of conditioning, 100 µl of medium was taken, mixed with 100 µl Chloroform, vortexed for 20 seconds and spun down at 15,000 rpm for 15 minutes at 4 degree. The aqueous phase was directly used for lactate measurement according to manufacturer’s protocol using the Roche D-Lactic acid/L-Lactic acid kit (#11112821035). Lactate concentration was then normalized to protein concentration of the cells the medium was taken from. Samples were measured in biological triplicates unless stated otherwise.

### Oxygen measurement

Cells were trypsinized, counted (~5 × 10^6^ cells/chamber) and resuspended in respiration medium (MiR05: 0.5 mM EGTA, 3 mM MgC_l2_*6H_2_O, 60 mM K-Lactobionate [lactobionic acid is dissolved in H_2_O, and pH is adjusted to pH 7.4 with KOH], 20 mM Taurine, 10 mM KH_2_PO_4_, 20 mM HEPES, 110 mM sucrose, 1 g/L fatty acid–free BSA) supplemented with 0.5 mM carnitine). Cells were added into the oxygraph chambers (final volume is 2.1 mL). Basal (intact) respiration was recorded. Then, cells were permeabilized by injecting 3 µL 4 mM digitonin solution into the chamber (final concentration is ~5.7 µM). After the signal stabilized, 5 μM palmitoyl-CoA was added to the chamber. Fatty acid β-oxidation was measured in the presence of complex I substrates, electron transfer flavoprotein (ETF) substrates, and ADP (10 mM proline, 10 mM pyruvate, 5 mM malate, 5 mM glutamate, 2 mM ADP, and 15 mM glycerol-3-phosphate). After recording the values, etomoxir (300 µM) was injected to inhibit fatty acid transport into mitochondria via CPT I, thereby inhibiting β-oxidation. Lastly, residual oxygen consumption (ROX) was measured by inhibiting complex III with antimycin A. All values were corrected for ROX and normalized by cell number. β-oxidation was calculated by subtracting etomoxir-resistant respiration from respiration in the presence of all substrates (n = 4).

### ATP measurement

After knockdown, cells were lysed and ATP was measured according to manufacturer’s protocol using CellTiter-Glo from Promega (#G7572).

### RNA and quantitative RT-PCR

RNA was isolated using TRIZOL reagent (Ambion, 15596018). Reverse transcription was done with RevertAid Premium Reverse Transcriptase (Thermo Scientific, EP0732). Q-PCR was done with Maxima SYBR Green/ROX master mix (Thermo Scientific, K0223). Genes were normalized to Rplp0 or mRpl13a levels.

### qPCR Oligos

**Table:** 

*qPCR - oligos*	
GCAGATCTTGAGGTTACGG	Rpl13a
TTGGTCTTGAGGACCTCTG	Rpl13a
AGATGCAGCAGATCCGCAT	Rplp0
GTTCTTGCCCATCAGCACC	Rplp0
GCTGAGGTACCAGTACTTGG	Crebl2
CCACTGCTTGTACATTTCCAG	Crebl2
GAGCCAGGTCCACATAGATG	Crebrf
TTGAAAGGCAAAGCAGGAGT	Crebrf
GTCCATTCCCTTCTCAGGG	Bloc1s1
AGTCTTCACGCACTTATTACTG	Bloc1s1
CCTTCAGATTGTTGCACGG	Cxcl12
CCAGGTACTCTTGGATCCAC	Cxcl12
ATCATCTGCTACCTGGACC	Fbxo36
GTCACTCTTGCACAACTTCTC	Fbxo36
AAACCTCCCGTTTGAATTACAG	Ing4
ATTTCAGCCTTCAGGTCCTC	Ing4
AGGAGGGCAAAGGAGTGTTT	Pfkp
TTGGCAGAAATCTTGGTTCC	Pfkp
TCCATGTGACCATGAGGAAA	Hif1a
CTTCCACGTTGCTGACTTGA	Hif1a

### Microarray analysis

TSC2−/− MEFs were treated with control Renilla Luciferase or Crebl2 siRNA #1 for 2.5 days. Then cells were treated with either EtOH or 20 nM rapamycin for 12 hours. RNA was isolated using TRIZOL, labelled using the Illumina TotalPrep RNA Amplification Kit (life technologies) and hybridized on an Illumina Mouse (WG-6_V2)-Microarray. Data were normalised using quantile normalisation with R using function normalize.quantiles from Bioconductor package “preprocessCore”.

### RNA-Seq

RNA was isolated using the RNAeasy Kit (Qiagen, #74104) and treated with RNA-free DNAse (Qiagen). Library preparation was done using the TruSeq® Stranded Total RNA Library Prep Kit (#20020597). Libraries were sequenced on a HiSeq 2000 v4 Sequencer (50 bp single-read). Resulting reads were uploaded and analyzed using the usegalaxy.org website^33^. For quality and adapter trimming we used Trim Galore! on default settings. Resulting filtered reads were aligned to the mouse genome using TopHat on default settings. The resulting BAM files were then subjected to htseq-count in order to count how many reads were in each gene. Resulting read counts per gene were then normalized to total read counts per sample by converting read per transcript into counts per million reads for each transcript (cpm) before further analysis.

### Data and analyses

Statistical significance in the figures was calculated using Student’s t tests.

## Supplementary information

Supplementary Information

Supplementary Table 1

Supplementary Table 2

Supplementary Table 3

Supplementary Table 4

Supplementary Table 5

Supplementary Table 6

Supplementary Table 7

## Data Availability

All microarray and RNA-seq data are available at NCBI Geo with accession number GSE134247.
