# Supplementary material for: Crebl2 regulates cell metabolism in muscle and liver cells
Source: Sci Rep. 2019 Dec 27;9:19869. doi: 10.1038/s41598-019-56407-w (PMC6934747; doi:10.1038/s41598-019-56407-w)

Supplementary Information

Crebl2 regulates cell metabolism in muscle and liver cells

Marcel Tiebe, Marilena Lutz, Deniz Senyilmaz Tiebe, and Aurelio A. Teleman

Contents

1. Supplementary Table Legends p.1

2. Legends for Supplementary Figures p.2

3. Supplementary Figures p.4

Supplementary Table Legends

Suppl. Table 1: Genes induced 2-fold by 12 h rapamycin treatment ($p<0.05$) in TSC2-/- MEFs treated with either control Renilla Luciferase siRNA or Crebl2 siRNA.

Suppl. Table 2: Gene ontology for genes listed in main Figure 1B.

Suppl. Table 3: Genes at least 2-fold upregulated ($p<0.05$) by Rapamycin in C2C12 myoblasts.

Suppl. Table 4: Genes at least 2-fold downregulated ($p<0.01$) by Crebl2 siRNA in C2C12 myoblasts.

Suppl. Table 5: Genes at least 2-fold upregulated ($p<0.01$) by Crebl2 siRNA in C2C12 myoblasts.

Suppl. Table 6: Genes at least 2-fold downregulated ($p<0.01$) by Crebl2 siRNA in Hepa1-6 cells.

Suppl. Table 7: Genes at least 2-fold upregulated ($p<0.01$) by Crebl2 siRNA in Hepa1-6 cells.

Legends for Supplementary Figures

Supplementary Figure 1: Cellular localization of Crebrf and Crebl2.

(A) Expression of 3xHA-Crebrf in $TSC2^{-/-}$ MEFs. 3xHA-Crebrf shows nuclear and/or cytoplasmic localization, and this varies from cell to cell. FITC - anti-HA, blue - DAPI, red - Rhodamine-Phalloidin. Scale bar = 10μm.

(B) Expression of 3xHA-Crebrf in 293T HEK cells. 3xHA-Crebrf is predominantly cytoplasmic with some nuclear staining. FITC - anti-HA, blue - DAPI, magenta - Cy5-Phalloidin. Scale bar = 10μm.

(C) Expression of GFP-Crebrf in 293T HEK cells. GFP-Crebrf is predominantly nuclear. green - GFP signal, blue - DAPI, magenta - Cy5-Phalloidin. Scale bar = 10μm.

(D) Expression of V5-Crebl2 in $TSC2^{-/-}$ MEFs. V5-Crebl2 is predominantly nuclear and forms small speckles within the nucleus. green - anti-V5, blue - DAPI, red - Rhodamine-Phalloidin. Scale bar = 25μm.

(E) Expression of HA-Crebl2 in TSC2$^{-/-}$ MEFs. HA-Crebl2 localization varies between cells and is either cytoplasmic or nuclear. green - anti-HA, blue - DAPI, red - Rhodamine-Phalloidin. Scale bar = 25µm.

(F) Expression of myc-Crebl2 in TSC2$^{-/-}$ MEFs. myc-Crebl2 is nuclear and forms large nuclear speckles. green - anti-myc, blue - DAPI, red - Rhodamine-Phalloidin
Scale bar = 25µm.

Supplementary Figure 2: Crebrf induction upon rapamycin treatment and knockdown efficiency of Crebl2 in C2C12 and Hepa1-6 cells.

(A) mRNA levels of Crebrf in C2C12 and Hepa1-6 cells treated with 20nM rapamycin for 14 and 12 hours, respectively.

(B) and **(C)** Knockdown of Crebl2 with siRNA #1 of Crebl2 for 2.5 days in C2C12 cells (B) or 3 days in Hepa1-6 cells (C).

Error bars: SD ***ttest<0.001

Supplementary Figure 1

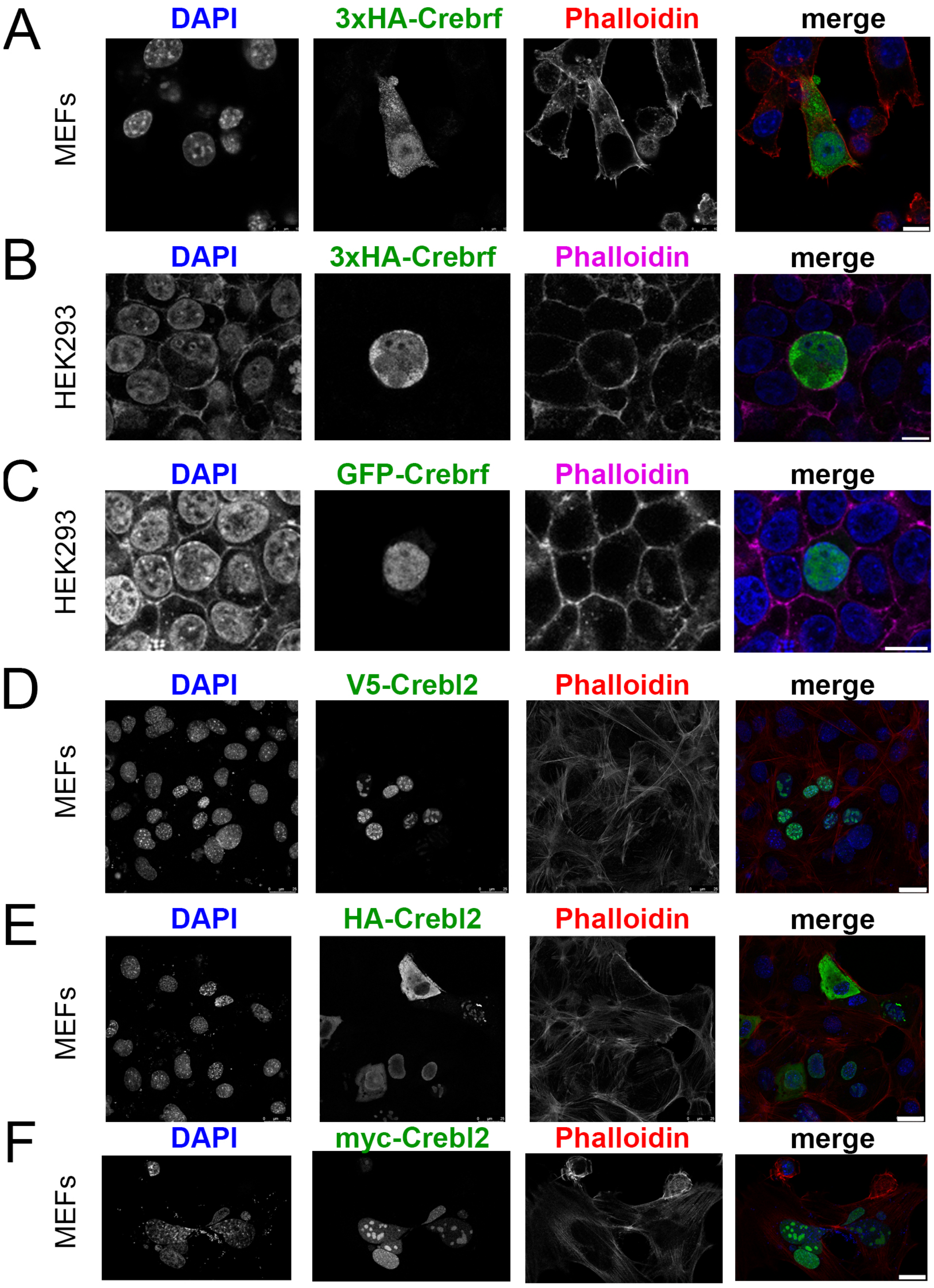

Supplementary Figure 2

A

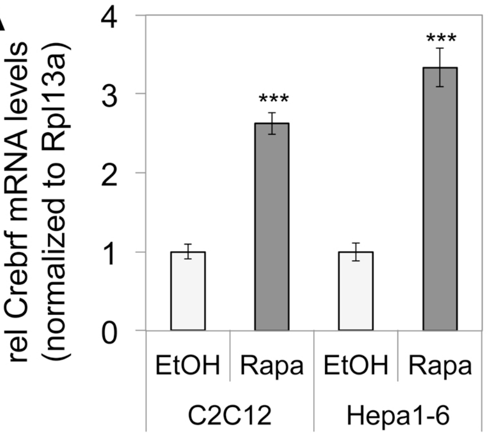

B

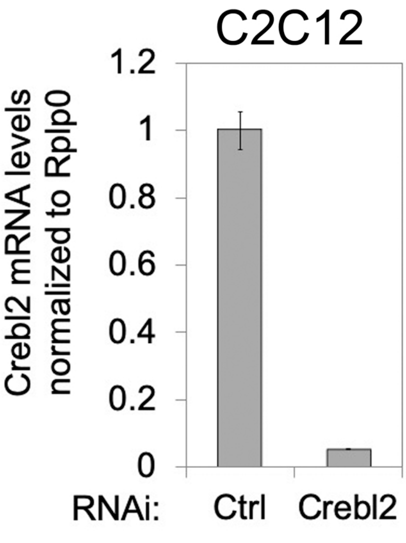

C

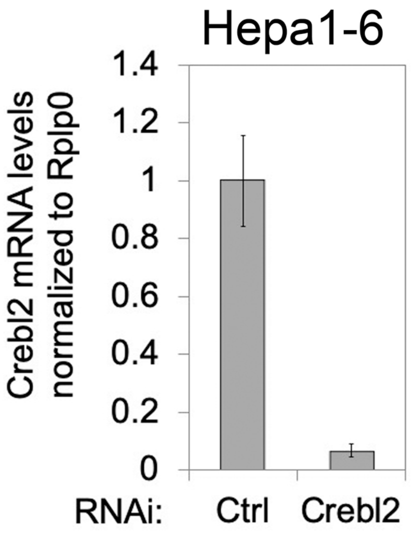

Supplement: Supplementary file 1 — Supplementary Information [file 41598_2019_56407_MOESM1_ESM.pdf]
